# Drug use and associated factors in a North Eastern region of Tanzania: a cross-sectional study

**DOI:** 10.11604/pamj.2022.43.70.35059

**Published:** 2022-10-11

**Authors:** Harrieth Peter Ndumwa, Castory Munishi, Jackline Eugene Ngowi, Belinda Jackson Njiro, Mangaro Mabusi, Sanita Suhartono, Anja Busse, Giovanna Campello, Giovanna Garofalo, Pietro Cipolla, Cassian Nyandindi, Omary Ubuguyu, Bruno Sunguya

**Affiliations:** 1 School of Public Health and Social Sciences, Muhimbili University of Health and Allied Sciences, P.O. Box 65001, Dar es Salaam, Tanzania,; 2 Aga Khan Hospital, P.O. Box 2289, Dar Es Salaam, Tanzania,; 3 United Nations Office on Drugs and Crime, A 1400 Vienna, Austria,; 4 Association Casa Famiglia Rosetta, Caltanissetta, Sicilia, Italy,; 5 Drug Control and Enforcement Authority (DCEA), P.O. Box 80327, Dar es Salaam, Tanzania,; 6 Directorate of Curative Services, Ministry of Health, P.O Box 743, Dodoma, Tanzania

**Keywords:** Knowledge, attitude, practice, people who use drugs (PWUDs), people who inject drugs (PWIDs)

## Abstract

**Introduction:**

Tanzania has experienced an increase in the number of people who use drugs (PWUDs) and people who inject drugs (PWIDs). Understanding the characteristics of PWUDs is crucial to addressing the increasing burden of drug use in Tanzania. This study was set to examine drug use and its related factors among PWUDs in a North Eastern region of Tanzania.

**Methods:**

a cross-sectional study conducted among 481 PWUDs in Tanga region, Tanzania. R statistical language was used for analysis and plotting. Logistic regression was performed to establish associations between knowledge and practice scores with drug use. A p-value of < 0.05 was considered statistically significant.

**Results:**

people who inject drugs comprised mostly of male (97.5%) and those with primary level education (71.1%). About three in four PWUDs had poor knowledge and practices towards drug use. Factors associated with adequate knowledge and practices towards drug use included residing in urban setting (aOR: 0.47, 95% CI; 0.29 - 0.74, p=0.001) while low level of education and use of drugs for less than 10 years were independent predictors for poor practices.

**Conclusion:**

drug use poses a significant threat among male and individuals with low education in Tanga region. Poor knowledge and practices towards drug use was more pronounced among rural and lowly educated PWUDs. Owing to variabilities of predictors, tailored and innovative interventions are needed to curb this growing drug use and associated effects in Tanga and other settings with similar contexts.

## Introduction

Drug use is a global problem and had contributed to about half a million deaths in 2019 alone, chiefly among the young populations [1]. The burden is set to hit 40% in Africa by 2030, unprecedented level compared to the global estimate of 11% in the 2021 [1]. Tanzania is no exception to such trend, with an observed rapid increase of number of people who use drugs (PWUD) and people who inject drugs (PWID) over the past one decade [2]. Tanzania has between 25,000 to 50,000 PWIDs [3]. Such burden is uneven between regions and is the highest among its major cities. Tanga region has about 25 hotspots of people who use drugs (PWUD) with around 5000 males and 190 females engaging in this high risk behavior [4].

The increase in the burden of drug use in Tanzania like in other settings has posed both health and development challenges, subjecting the affected into cycles of infectious diseases, lost productivity, inefficiency, and rise in crime [5,6]. Initiatives to increase the access to services for PWUDs and PWIDs, such as the establishment of treatment centers offering Opioid agonist maintenance and drug use primary prevention activities are being conducted in the country [7]. The effect in terms of uptake of interventions and response thereof highly depends on their knowledge, attitude, practices towards use of drugs. Understanding these and other characteristics of drug use in line with critical factors associated thereof remains crucial to the success of such interventions.

Owing to variabilities of drug use patterns and characteristics thereof, it is essential to establish levels of knowledge, attitudes, practices, and other contexts under which people who use drugs are living. This in turn will affect significantly the science of uptake of any interventions including targeted interventions for PWUDs and PWIDs, through tailoring them. This study assessed the knowledge, attitude, practices and factors associated with drug use in Tanga urban and rural areas. The findings of this study provide recommendation for suitable innovative approaches for primary prevention targeted particularly in this setting where the burden of drug use is rapidly growing.

## Methods

**Study design and setting:** this community based cross-sectional study was conducted in Tanga, a North Eastern region of Tanzania bordering Kenya with an area of 474 square kilometers and a population of 2,509,400 people [8]. It is an important drug supply route from and to the global markets through open sea and road networks, as expected therefore, the burden of drug use is high with PWID and PWUD; 540 and 190 respectively [6]. The deep port of Tanga is also a home for big heroin trade dealers in Tanzania [9]. Moreover, Tanga has a unique pattern of drug use taking place in the households as contrary to other regions in the country where drug use and dealing occurs in hidden places found in streets [10]. The two districts of Tanga region, Tanga City and Muheza were selected to represent urban and rural settings respectively.

**Study population and selection:** we included PWUDs and PWIDs from Muheza and Tanga district with at least 18 years of age. People who use drugs and people who inject drugs who were found intoxicated or suspected to be under influence of drugs at the time of data collection were excluded.

**Sampling and sample size:** a minimum sample size of 465 was calculated using the proportional formula for calculating sample size (Kish and Leslie formula) [11]. The proportion for substance use of 50% and a standard error of 5% were used for calculation owing to lack of recent population-wide evidence. We used convenient sampling technique to recruit participants in this study. This data collection method was employed due to the challenges of reaching PWUD in data collection sites, the drug hotspots. Participants who met the inclusion criteria from both districts, Tanga city and Muheza and consented to participate in the study were enrolled.

**Measurements and tools:** structured interviewing forms were used to obtain data on knowledge, attitude and practice towards drug use. The interviewing forms consisted of five sections, based on the specific objectives and, had a total of 39 questions, 34 close-ended and 5 open-ended questions. Participants were interviewed individually and in privacy to ensure confidentiality. Each interview took approximately 20 minutes to complete. The first section consisted of 10 questions on demographic information, the second section comprised of 7 questions on general knowledge on substance use, the third section had 6 questions on attitude towards substance use and the last sections had a total of 16 questions on alcohol and drug use practices. The tool was developed in English language and then translated into Swahili; a national language spoken by many in the region. A pilot study was conducted to pre-test the tools before the actual data collection, using simulated participants. Necessary corrections and modification of the instruments were then made to obtain the final version that was used during the actual data collection.

**Data collection:** data collection was conducted by trained research assistants simultaneously in both districts in October and November 2020 until the allocated sample size was reached. The research assistants were trained before hand on the data collection tool, ethical conduct, and together reviewing the tool for validation. The interviews took about 30 minutes and were conducted 500 meters from the drug hotspots to ensure privacy and confidentiality.

**Study variables:** the study had three main outcome variables, knowledge, attitude and practices. The knowledge assessment section had a mean (standard deviation) score of 11.98 (±3.81), we used a cutoff point of 13 to categorize participants as having good or poor knowledge. In assessing attitude, the mean (standard deviation) score was 20.48 (±3.51), a cutoff point score of 15 was used to categorize participants into good and poor attitude and, mean (standard deviation) for practice score was 6.04 (±1.34) with a score of 6 as a cutoff point between good and poor practices towards drug use. The last variable was socio-economic factors affecting knowledge and practice towards drug use.

**Data management and analysis:** data coding, data entry and cleaning were completed using R statistical software. R statistical language was used for analysis and plotting. The obtained data was summarized into frequency tables, graphs and cross tabulations. Descriptive analysis was performed and presented in frequencies, percentages, means and their corresponding standard deviations. Bivariate and multivariate logistic regression analysis on socio-economic variables affecting knowledge and practice scores were calculated. A p-value of <0.05 was considered to be statistically significant.

**Ethical approval and consent to participate:** ethical clearance was obtained from the MUHAS Institutional Review Board (IRB), reference number Ref. No.DA.282/298/01.C/, MUHAS-REC-12-2020-438. Permission to conduct the study was obtained from the Permanent Secretary of the Ministry of Health, Community Development, Gender, Elderly and Children, the Regional Medical Officer of Tanga Region and the District Medical Officers from Tanga City and Muheza District. Additionally, permission to collect data from Bombo MAT clinic was further obtained from the Medical Officer in Charge and head of the MAT unit. Assent to participate in the study was sought from the eligible study candidates.

## Results

**Participant´s characteristics:** a total of 481 participants were enrolled in the study from both Tanga city and Muheza district, the response rate was 100%. The age range of the participants was between 20 and 72 years, with a mean age (SD) of 37.72 ± 7.89. Majority of the participants were males 97.5% (469/481) and single 54.1% (260/481). About three quarter of the participants 71.1% (342/481) reported having attained primary level of education. More than half of the participants 59.8% (287/481) had used drugs for the first time at the age of 15 to 24 years, with a mean age (SD) of 24.29 ± 5.96 and, the majority 58.9% (282/481) had used drugs for the period of more than 10 years (Table 1).

**Table 1:** socio-demographic characteristics of the study participants stratified by study area n (%)

Characteristic	Total (N=481)	Muheza (N=121)	Tanga (N=360)	P-value
**Sex**				
Male	469 (97.5)	121 (100)	348 (96.7)	0.090
**Age groups**				
18 - 25 years	24 (5.0)	9 (7.4)	15 (4.2)	0.556
25 - 34 years	182 (37.9)	45 (37.2)	137 (38.1)	
35 - 44 years	202 (42.1)	40 (41.3)	153 (42.5)	
45+ years	72 (15.0)	17 (14.0)	55 (15.3)	
**Marital status**				
Single	260 (54.1)	68 (56.2)	192 (53.3)	0.379
Married	91 (18.9)	18 (14.9)	73 (20.3)	
Widowed	14 (2.9)	2 (1.7)	12 (3.3)	
Divorced	116 (24.1)	33 (27.3)	83 (23.1)	
**Education**				
No education	23 (4.8)	1 (0.8)	22 (6.1)	0.032*
Primary level	342 (71.1)	85 (70.3)	257 (71.4)	
Secondary and above	116 (24.1)	35 (28.9)	81 (22.5)	
**Can read and write**				
Yes	438 (91.1)	113 (93.4)	325 (90.3)	0.393
**Has a home**				
No (homeless)	97 (20.2)	28 (23.1)	69 (19.2)	0.417
**Residential status**				
Alone	54 (12.9)	15 (15.5)	39 (12.1)	0.086
With my children	16 (3.8)	4 (4.1)	12 (3.7)	
With family	170 (40.5)	27 (27.8)	143 (44.3)	
With friends	30 (7.1)	10 (10.3)	20 (6.2)	
With wife	27 (6.4)	9 (9.3)	18 (5.6)	
With parents	123 (29.3)	32 (33.0)	91 (28.2)	
**Age first used drugs**				
< 15 years	19 (4.0)	3 (2.5)	16 (4.5)	0.211
15 - 24 years	287 (59.8)	65 (53.7)	222 (61.8)	
25 - 34 years	156 (32.5)	48 (39.7)	108 (30.1)	
35 + years	18 (3.8)	5 (4.1)	13 (3.6)	
**Used drugs for > 10 years**				
Yes	282 (58.9)	64 (52.9)	218 (60.9)	0.150

*Factors that were statistically significant in comparing the two study settings (p<0.05)

**Participants´ knowledge on drug use:** the mean (SD) knowledge score of the study participants was 11.98 (±3.81). Almost three quarters 72.7% (352/481) of the study participants had poor knowledge (a score of less than or equals 13). The prevalence of having good knowledge among participants from the rural area (Muheza), and from the urban area (Tanga City) were 41.1% (53/121) and 22.8% (85/360) respectively showing a statistically significant association between the specific geographical location and knowledge on drug use, p < 0.001 (Table 2). Majority of the participants 88.77% (419/481) responded to have known what drug use was. Most of the participants mentioned drugs causing drug use disorders to be opioids 79.2% (381/481), marijuana 68.4% (329/481), cocaine 52.6% (253/481) and sedatives 29.3 (141/481). Other drugs such as tobacco 24.7% (119/481), alcohol 18.1% (87/481), inhalants 8.1% (39/481), amphetamines 3.5% (17/481) and hallucinogens 3.1% (15/481) were mentioned by a few participants as indicated. The effects of illicit drug use mentioned by the majority of the participants included; poor health 54.3% (261/481), crimes 43.9% (211/481), stigma 37.8% (182/481) and HIV infection 22.5% (108/481). Most of the participants mentioned Methadone 81.9% (394/481) and rehabilitation 27.9% (134/481) as the treatment options for drug use disorders. Only a few participants knew about other treatment modalities, cognitive behavioral therapy 6.9% (33/481), family therapy 3.3% (16/481) and other alternative therapies (medications) 0.6% (3/481).

**Table 2:** participants' knowledge, attitude and practices towards drug use stratified

Characteristic	Total (N=481)	Muheza (N=121)	Tanga (N=360)	P-value
**Knowledge score (mean (SD)**	12.0 (3.8)	13.4 (4.7)	11.5 (3.4)	
**Knowledge level**				
Good knowledge	129 (27.3)	48 (41.4)	81 (22.8)	< 0.001
Poor knowledge	352 (72.7)	68 (58.6)	275 (77.2)	< 0.001
**Attitude score (mean (SD)**	20.5 (3.5)	18.6 (3.4)	21.1 (3.3)	
**Attitude level**				
Good attitude	456 (94.2)	107 (87.4)	349 (96.6)	<0.001
Poor attitude	25 (5.8)	14 (12.6)	11 (3.4)	0.001
**Practices score (mean (SD)**	6.04 (1.34)	6.4 (1.0)	5.91 (1.4)	
**Practice level**				
Good practices	110 (22.9)	13 (10.7)	97 (26.9)	<0.001
Poor practices	371 (77.1)	108 (89.2)	263 (73.1)	<0.001


• All factors show statistical significance (p<0.05)

**Figure 1:**
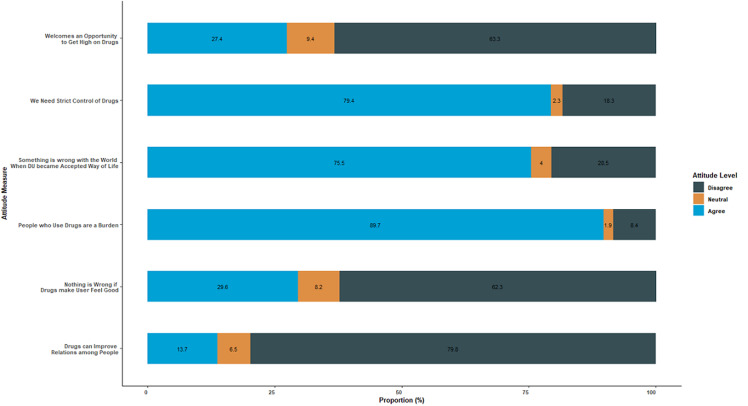
participants' responses on attitude towards drug use

**Participants´ attitudes towards drug use:** the mean (SD) attitude score was 20.48 (±3.51). Majority of the study participants 94.2% (409/481) had good attitude in terms of looking at drug use as a problem (score ≥15). Urban setting (Tanga City) had a slightly higher number of participants, 96.6% (349/360) with good attitude as compared to the rural setting (Muheza) 87.4% (107/121), showing that PWUDs residing in the city have a better attitude as compared to those in rural areas, with a statistically significant difference, p<0.001 (Table 2). Participants´ responses on different attitude questions are described in Figure 1.

**Participants practice towards drug use:** the mean (SD) practice score towards drug use among participants was 6.04 (±1.34). More than three quarters of the study participants 77.1% (371/481) had poor practice towards drug use (score ≥6). The rural setting (Muheza) had a greater proportion of participants with poor practice 89.3% (108/121) as compared to the number of participants 73.1% (263/360) from the urban setting (Tanga City) with a statistically significant association p <0.001 (Table 2). We used five questions to assess practice towards drug use. One of the questions asked how often has a participant used illicit drugs. Majority of the participants, 84.62% (407/481) reported to have used illicit drugs about four or more times a week with very few 4.16% (20/481) reporting to have used illicit drugs monthly or less. About three-quarter of the participants reported to be using heroin 72.3% (348/481), marijuana 70.1% (337/481) and a third reported to use cocaine 30.1% (145/481) with only one participant reporting to have ever used amphetamine. Majority of the participants 97.51% (467/481) reported not to inject illicit drugs. Another question asked if participants have ever shared needles and syringes with others, majority 99.38% (478/481) reported to have not shared needles and syringes with others.

***Factors associated with knowledge among study participants:*** on bivariate analysis, residence was found to be the only factor showing independent association with knowledge among PWUDs. PWUDs in the urban area (Tanga City) were 53% less likely to have poor knowledge (aOR: 0.47, 95% CI; 0.29 - 0.74, p=0.001) as compared to those in the rural area (Table 3).

**Table 3:** logistic regression analysis on socio-economic variables affecting knowledge and practice scores

Variable	Knowledge		Practice	
Sex	aOR (95% CI)	p-value	aOR (95% CI)	p-value
Female	1			
Male	0.64 (0.17 - 3.06)	0.525	1.07 (0.26 - 3.81)	0.915
**Age groups**				
18 - 25 years	1		1	
25 - 34 years	0.57 (0.21 - 1.57)	0.263	1.11 (0.31 - 3.48)	0.861
35 - 44 years	0.47 (0.15 - 1.47)	0.186	1.82 (0.45 - 6.56)	0.373
45+ years	0.59 (0.16 - 2.25)	0.439	2.46 (0.52 - 10.8)	0.238
**Marital status**				
Single	1		1	
Married	0.55 (0.28 - 1.03)	0.071	0.55 (0.30 - 1.01)	0.050
Widowed	1.14 (0.28 - 3.95)	0.842	0.65 (0.18 - 2.70)	0.522
Divorced	1.11 (0.65 - 1.87)	0.695	0.75 (0.42 - 1.35)	0.330
**Education**				
No education	1		1	
Primary level	1.44 (0.50 - 5.22)	0.531	3.26 (2.16 - 8.27)	0.013*
Secondary +	1.82 (0.59 - 6.92)	0.333	2.00 (0.72 -5.48)	0.177
**Has a home**				
Yes	1		1	
No	0.87 (0.49 - 1.51)	0.635	1.03 (0.57 - 1.92)	0.935
**Age first used drugs**				
< 15 years	1		1	
15 - 24 years	2.00 (0.59 - 9.32)	0.312	1.80 (0.63 - 4.99)	0.258
25 - 34 years	2.59 (0.70 - 12.8)	0.188	1.99 (0.62 - 6.25)	0.236
35 + years	2.77 (0.45 - 19.4)	0.282	0.50 (0.10 - 2.58)	0.400
**Used drugs for > 10 years**				
No	1		1	
Yes	1.03 (0.58 - 1.83)	0.925	0.49 (0.26 - 0.91)	0.026*
**Location**				
Muheza district	1		1	
Tanga city	0.47 (0.29 - 0.74)	0.001*	0.36 (0.18 - 0.66)	0.002*

* Factors that were statistically significant

***Factors associated with practices among study participants:*** participants with primary level of education, were more than three times likely to have poor practices towards drug use (aOR: 3.26, 95% CI; 2.16 - 8.27, p=0.013) as compared to those with no education. Participants who had used drugs for more than 10 years were 50% less likely to have poor practices (aOR: 0.49, 95% CI; 0.26 - 0.91, p=0.026) as compared to those who used drugs for less than 10 years. Moreover, participants in the urban area (Tanga City) were 74% less likely to have poor practices (aOR: 0.36, 95% CI; 0.18 - 0.66, p=0.002) as compared to those in the rural area (Table 3).

## Discussion

This study assessed characteristics, knowledge, attitude and drug use practices among PWUD in Tanga, Tanzania. The current evidence shows that more than half of the participants 59.8% (287/480) had used drugs for the first time at the age of 15 to 24 years and the majority 58.9% (282/481) have used drugs for more than 10 years of their life. Nearly three quarters 72.7% (352/481) of the study participants had poor knowledge, majority 94.2% (409/481) had good attitude and about three quarters 77.1% (371/481) had poor practice towards drug use. Residing in urban setting was significantly associated with good knowledge and practices towards drug use. Level of education and use of drugs for more than 10 years were independent predictors for poor practices among study participants. The large proportion of participants (97.5%) in this study were males similar to the current understanding that men are more likely to use drugs compared to women [12]. The low proportion of female participants in this study, reflects the existing perceived stigma and disapproval of drug use leading to possible under reporting [13]. The findings could also be a reflection of low prevalence of drug use among females as noted elsewhere [14,15].

Our study found that just above half of the participants were single, with only one fifth of participants living with their partners; these findings are consistent with other studies which reported the quality of the marital relationship to be important in predicting drug use [16]. For PUWDs, drug use is their main priority, in which they spend most of their time on drugs other than building families or relationship [17], moreover, drug use is highly discouraged in the society and majority of people who use drugs and with drug use disorders engage in criminal activities such as theft which segregate them from the society and social relationships [18]. Family relationships such as marriage or having a close relationship appears to discourage drug use and to prevent relapse during treatment [19].

This study found that more than half of participants had started using drugs during teen age, adolescence and young adulthood. Similar to our findings, evidence has indicated that this group of people are likely to experiment new things and are highly influenced by peer pressure, hence marking this age group as a critical period for initiation of drug use [20-22]. It is also explained further to be as a result of curiosity and seeking personal awards such as to become fashionable and socially acceptable but also linked to delinquent behaviour [23]. However, initiation of illicit drug use in this period is associated with continued drug use during adulthood, development of dependence, polydrug use and many other adverse social, educational and mental health outcomes [23,24].

The mean knowledge score in this study was found to be 11.98 (±3.81) with about three quarters of the participants having poor knowledge. The mean knowledge score is twice as high as that reported in a study done in India which assessed substance use knowledge among people with tobacco and alcohol use disorders, 5.04 ± 1.27 and 4.88 ± 1.22 respectively [25]. However, another study conducted in Jordan had shown much higher level of knowledge among the middle aged population group [26] and among health sciences´ students [27,28]. The differences observed can be explained by the diversities of the tools used for assessment.

The mean (SD) for attitude score in this study was found to be 20.48 (±3.51) with majority of the study participants having good attitude towards drug use. This score is comparatively higher than the attitude score among people with tobacco and alcohol use disorders in India which was found to be 1.42 ± 1.71 and 1.65 ± 1.69 respectively [25]. However, other studies have reported much higher attitude scores which can be explained by involvement of relatively younger and educated participants in their studies [8,29]. Good attitude score among participants from the urban area (Tanga City) is similar to other studies and can be explained by presence of awareness programs and accessibility of drug use treatment services in the urban setting as compared to the rural area [29,30]. The current evidences show that more than three quarters of the study participants stipulated poor practice towards drug use which is in keeping with a study done in India which reported a low practice score among people with alcohol and tobacco disorders [25].

Residing in urban setting was significantly associated with having good knowledge and practices towards drug use. These findings are in line with other studies and could mainly be explained by the easy access to correct information on drug use, availability of treatment and rehabilitation centers and less stigma in urban areas as compared to the rural areas [29,30]. Surprisingly, participants with primary level of education in this study had poor practices compared to those who have not been to school, contrary to studies done in the US [31] and UK [32]. Shorter periods of using drugs, less than 10 years in this study was also found to be associated with poor practices towards drug use. These findings are inconsistent with a study done in the US [33] which explains that prolonged use of drugs increases vulnerability and augments risky drug use [34].

**Strengths and limitations:** to the best of our knowledge, this is the first study done to assess knowledge, attitude, drug use practices and associated factors among PWUDs and PWIDs in Tanga region, Tanzania. The study has included a representation of both urban (Tanga City) and rural (Muheza) areas. Furthermore, despite having a unique kind of drug use pattern at household level which goes further to affecting the younger population in Tanga, not much has been done to represent and explain this pattern.

Besides the strengths of this study, a number of limitations were noted. Provision of socially desirable answers as a result of fear may have resulted in response bias as drug use is a sensitive subject and illegal in Tanzanian communities. During data collection, participants were explained in details on the purpose of the study and were assured of confidentiality of the information they provided including not collecting individual names or any kind of personal identification information. Additionally, considering the nature of the study design, recall bias could contribute as one of the limitations since some information necessitated the participants to recall events of the past in which case, they might not be as accurate in recalling hence increasing the risk of documenting unreliable information. Further, the study was done among PWUDs in one region of the country where social and cultural factors are different from other settings making it challenging to generalize the findings for all PWUDs in Tanzania.

## Conclusion

Findings from this study shows poor knowledge and practices, however good attitude towards drug use among the PWUDs and PWIDs in Tanga region. Most of the participants had started drug use during teen age, adolescence and young adulthood which is concerning and necessitates the need of youth friendly services and application of innovative methods to raise awareness among youths and adolescents on the immediate and long-term effects of drugs use. Area of residence, level of education and duration of drug use were found to be associated with knowledge and practices towards drug use. Awareness should also be raised on the various treatments available for drug use and should target more PWUDs/PWIDs in rural setting who appear to be less knowledgeable and to have poor practices.

**Funding:** the study was funded by the United States Bureau of International Narcotics and Law Enforcement Affairs US/INL through the UNODC´s implementing partner, the Association Casa Famiglia Rosetta as a sub-project of the project named “Awareness in Caring Community, Response preparedness and interventions supporting PWUD and other vulnerable groups” **conducted in Tanga Tanzania**.

**Disclaimer:** Anja Busse, Sanita Suhartono and Giovanna Campello are staff members of the United Nations. The views reflected in the articles are those of the authors and do not necessarily reflect the views of the United Nations.

### What is known about this topic

- Urban residency is associated with better level of knowledge, attitude and practices towards drug use compared to rural setting;
- Drug use normally starts during teen age, adolescence and young adulthood.

### What this study adds

- We found that PWUDs and PWIDs in Tanga Tanzania have poor knowledge and practices but have good attitude towards drug use;
- Shorter periods of using drugs, less than 10 years in this study was associated with poor practices towards drug use;
- To the best of our knowledge, this is the first study done to assess knowledge, attitude, drug use practices and associated factors among PWUDs and PWIDs in, Tanzania.
